# Long-term effects of sublingual immunotherapy (SLIT) in preventing the development of asthma in allergic rhinitis: Revision of 15-year follow-up^☆^

**DOI:** 10.1016/j.waojou.2026.101434

**Published:** 2026-07-28

**Authors:** Maurizio Marogna, Alessandro Massolo, Giovanni Passalacqua, Benedetta Bondi, Sara Fedele, Giorgio Walter Canonica, Diego Bagnasco

**Affiliations:** a Poliambulatorio Induno Olona, Induno Olona, VA, Italy; b Ethology Unit, Department of Biology, University of Pisa, Pisa, Italy; c Faculty of Veterinary Medicine, University of Calgary, Calgary, Alberta, Canada; d UMR CNRS 6249 Chrono-environnement, Université Franche-Comté, Besançon, France; e Respiratory and Allergy Clinic, IRCCS Policlinico San Martino, Genoa, Italy; f Department of Internal Medicine (DIMI), University of Genoa, Genoa, Italy; g Personalized Medicine, Asthma and Allergy, IRCCS Humanitas Research Hospital, Rozzano, Milan, Italy; h Department of Biomedical Sciences, Humanitas University, Pieve Emanuele, Milan, Italy

## Abstract

**Background:**

Sublingual immunotherapy (SLIT) has been shown to be effective in controlling the symptoms of allergic rhinitis and asthma, but few studies have assessed its long-term preventive effects on lower-airway inflammation and asthma onset.

**Objective:**

We sought to evaluate prospectively the long-term effects of SLIT on lower-airway function in patients with allergic rhinitis caused by house dust mites (HDM).

**Methods:**

We performed a post hoc analysis of a previously published prospective, open-label study involving patients with allergic rhinitis who were monosensitized to mites and followed for 15 years. All participants had bronchial hyperreactivity and were originally assigned to 4 groups receiving either pharmacological therapy alone or SLIT for 3, 4, or 5 years (SLIT 3, SLIT 4, and SLIT 5). Skin sensitization, methacholine reactivity, and respiratory function were evaluated annually during the winter months.

**Results:**

Seventy-eight patients were enrolled, and 59 completed the study. Over 15 years of observation, new sensitizations occurred in all participants in the control group but in fewer than one-quarter of patients who received SLIT for 3, 4, or 5 years (21%, 12%, and 11%, respectively). Among patients with rhinitis treated only with intranasal corticosteroids and systemic antihistamines, the development of asthma, defined as a decline in FEV to below 80% of the predicted value, was observed in 58% (7 of 12). In contrast, progression to asthma was significantly less frequent among patients with rhinitis treated with SLIT, regardless of treatment duration: 1 of 14 patients in the SLIT 3 group (7%), 1 of 16 in the SLIT 4 group (6%), and 1 of 17 in the SLIT 5 group (6%).

**Conclusion:**

In the long term, SLIT provided a significant clinical benefit in HDM-induced allergic rhinitis by reducing both the allergic march and asthma onset and by limiting the decline in lung function, as measured by FEV1.

## Introduction

In recent decades, our understanding of the pathogenesis of rhinitis, rhinosinusitis, and asthma, particularly the T2 phenotype,^1^ has improved substantially. In asthma, the focus has shifted from bronchial smooth muscle dysfunction (bronchospasm) to the inflammatory paradigm, encompassing T2 and non-T2 inflammation,^2^ and to the concept of airway remodeling.^3^ More specifically, airway remodeling can be defined as an alteration in the anatomical and functional architecture of the airways following histological changes, including basement membrane thickening, subepithelial collagen deposition, and hypertrophy and hyperplasia of fibroblasts and bronchial smooth muscle cells. If not adequately counteracted, these changes lead to irreversible bronchial obstruction.^4^ An important area of research is whether treatment can alter the natural history of the disease by interacting with and modifying these mechanisms. Various treatments have been used to control the cascade of biomolecular and cellular events leading to chronic inflammation, initially systemic and subsequently inhaled corticosteroids, followed by biological therapies and allergen immunotherapy (AIT). Among these treatments, only AIT fully meets the criteria for precision medicine^5^ and is often considered the earliest example of a precision-based approach to respiratory allergic diseases such as rhinitis and asthma. It is therefore included in both the Allergic Rhinitis and its Impact on Asthma (ARIA)^6^^,^^7^ guidelines for rhinitis management and the Global Initiative for Asthma (GINA) guidelines for asthma.^8^

Several studies have investigated the close relationship between rhinitis and the development of asthma. One study using bronchial biopsies in pediatric patients demonstrated that signs of airway remodeling were already present at an early age, even in patients with rhinitis alone.^9^ The well-established pathophysiological and inflammatory relationship among asthma, rhinitis, and rhinosinusitis makes it possible to assess whether therapies used for upper-airway disease, such as allergen immunotherapy for rhinitis, provide not only disease control but also a disease-modifying effect on asthma.^10^ On the basis of these observations, we sought to determine the extent to which AIT may act as a biological disease modifier during progression from rhinitis to asthma. We therefore analyzed a functional index, forced expiratory volume in 1 second (FEV1), using data collected during a 15-year follow-up from 1991 to 2006. FEV1 was selected because several authors consider it an indirect biomarker of airway remodeling^11^ that is easily and reliably reproducible. The main objective of this study was therefore to reanalyze data from a previously published study by Marogna et al^12^ using a different and more specific statistical approach. We evaluated changes in FEV1 over 15 years in patients with rhinitis and confirmed airway hyperreactivity, comparing those who received standard therapy alone with those who also received AIT.

## Patients and methods

We conducted a post hoc analysis and statistical reevaluation using robust methods on the previously published study by Marogna et al. The original investigation was an open-label, controlled, partially randomized study with 4 parallel groups and included patients with allergic rhinitis and confirmed concomitant bronchial hyperreactivity.^12^ All patients were monosensitized to Dermatophagoides. At enrollment, none had clinical or functional signs of persistent asthma (FEV1 ≥ 80% of the predicted value). They were randomized to receive standard pharmacotherapy alone (control group) or in combination with sublingual immunotherapy (SLIT) targeting Dermatophagoides for 3, 4, or 5 years (SLIT 3, SLIT 4, and SLIT 5).

After the baseline evaluation, patients were randomly assigned to the control group (pharmacotherapy alone) or the SLIT group (SLIT plus pharmacotherapy). Patients receiving SLIT were then allocated according to their date of birth, specifically the 10-day period of the month in which they were born, to receive treatment for 3, 4, or 5 years. Assessments were conducted annually. Pulmonary function and skin prick tests were performed each year between September and February. The study ran from 1992 to 2007. The ethics committee approved the study but did not permit treatment blinding because of ethical concerns related to the duration of follow-up. The inclusion and exclusion criteria are summarized in Table 1.

**Table 1:** Inclusion/exclusion criteria used in Marogna M et al.^12^ Inclusion and exclusion criteria used to select patients with allergic rhinitis and bronchial hyperreactivity for the original study cohort

INCLUSION CRITERIA	EXCLUSION CRITERIA
Age 18–65 years, both sexes	Previous AIT
Allergic rhinitis without persistent asthma for >2 years	Severe nasal abnormalities
Monosensitization to HDM (skin test/RAST)	Polysensitization (skin test/RAST)
Normal spirometry (FEV1 ≥ 80% of predicted)	FEV1 ≤ 79% of predicted
Positive methacholine challenge	Pregnancy or psychiatric disorder
Nasal eosinophils ≥ 20% on nasal brushing	Severe systemic autoimmune disease

Rhinitis was clinically diagnosed based on the presence of the 4 classic symptoms: rhinorrhea, sneezing, nasal obstruction, and itching. Asthma was diagnosed based on episodes of wheezing, chest tightness, dyspnea, or cough that improved after inhaled salbutamol. Because the Global Initiative for Asthma guidelines were not yet available in 1991, patients were eligible if they had episodic asthma and normal FEV1 on spirometry. Skin tests were performed at baseline and annually thereafter, at the end of the winter season, in accordance with standardization guidelines^13^ and using a standard allergen panel (ALK-Abell, Milan, Italy) that included the major allergen sources.

Sublingual immunotherapy (SLIT) was administered as a glycerinated allergen extract in drops (Anallergo, Florence, Italy), taken once daily in the morning. The SLIT preparation was biologically standardized, expressed in RAST units per milliliter, and supplied in 5 vials of increasing concentration (100, 300, 1,000, 3,000, and 10,000 RAST U/mL). After the induction phase, patients received a maintenance regimen of 5 drops from the vial with the highest concentration (10,000 RAST U/mL), administered 3 times weekly throughout the year. The estimated mean annual cumulative dose was equivalent to 390 mg of Der p 1/Der p 2, nearly tenfold higher than that used for subcutaneous immunotherapy (SCIT).

Patients were instructed to monitor and record all adverse events in a personal diary and were provided with continuous telephone access to a physician for SLIT-related concerns. Treatment adherence was assessed by measuring the residual volume in returned vials and comparing it with expected use to calculate a percentage adherence index.

Concomitant pharmacological therapy was prescribed from September to February and included daily cetirizine (10 mg), intranasal cromolyn, and salbutamol as needed for asthma control (100–200 g). In addition, nasal beclomethasone (400 g/day), inhaled salmeterol (50 g twice daily), and inhaled beclomethasone (500 g twice daily) were used according to clinical judgment. These pharmacotherapies were selected in the pre-guideline era (1992), before publication of the GINA and ARIA recommendations. Treatment protocols were subsequently updated as guidelines evolved and newer inhaled or intranasal corticosteroids, such as budesonide and fluticasone, became available; these agents were used at the doses specified in their prescribing information.

Pulmonary function was assessed using a whole-body plethysmograph combined with a computerized spirometry system (Jaeger, Würzburg, Germany). Airway hyperresponsiveness was evaluated by methacholine bronchoprovocation testing with a dosimeter (Jaeger) that delivered aerosolized methacholine in response to the patient's inspiratory effort. Increasing cumulative doses of methacholine (30, 60, 120, 240, 390, 690, and 1290 g) were administered sequentially until FEV had decreased by ≥ 20% from baseline.^14^^,^^15^ Each participant underwent spirometry with a methacholine challenge annually at the end of the winter season (approximately February), after refraining from inhaled bronchodilator therapy for at least 12 hours.

## Statistical analysis

Differences in sex distribution among the 4 groups at baseline were assessed using the chi-square test, whereas differences in age and baseline FEV were evaluated using a two-way ANOVA with SLIT treatment and sex as fixed factors. The incidence of a decline in FEV to below 80% of the predicted value was compared across groups using Fisher's exact test because of the relatively small subgroup sizes.^16^

To evaluate the longitudinal effect of SLIT on respiratory function, we used generalized linear mixed-effects models (GLMMs), an approach well suited to the hierarchical data structure and repeated annual measurements within each participant. FEV was treated as a continuous dependent variable. TREATMENT and SEX were included as fixed effects, whereas AGE was entered as a continuous covariate to account for the potential effect of aging on lung function.^17^

To model within-participant correlation and interannual variability, PATIENT ID and YEAR were specified as random effects. Alternative random-effects structures (random intercepts alone versus random intercepts and slopes) were tested to identify the model that best represented the data structure. Parameters were estimated using maximum likelihood, allowing direct comparison of nested models.

Model selection was based on the Akaike information criterion (AIC) and likelihood-ratio tests using chi-square statistics. For all fixed effects, we report effect sizes and 95% confidence intervals to permit quantitative assessment of the magnitude and precision of the estimated associations. Clinical relevance was evaluated by interpreting effect sizes in the context of expected long-term changes in FEV among patients with allergic rhinitis and bronchial hyperreactivity.

All analyses were conducted in R version 4.3.1 (2023-06-16). GLMMs were fitted using the glmmTMB package (version 1.1.7). Baseline ANOVAs and model comparisons were performed using the anova() function. Figures were generated using ggplot2 (version 3.4.3).

Although the final sample size and subgroup sizes are limitations, this study was derived from a prospective real-world cohort with an exceptionally long follow-up (15 years), in which retention of approximately 75% of participants represents a methodologically robust outcome. Moreover, the cohort was highly homogeneous, comprising monosensitized patients with rhinitis and bronchial hyperreactivity; this reduced variability and increased statistical efficiency, partly offsetting the limited sample size. The observed between-group differences were large, consistent, and biologically plausible, suggesting genuine effects despite the small sample. Therefore, although this limitation must be acknowledged, we believe that the results retain interpretive robustness and value for hypothesis generation.

Compared with the original study, the new statistical analyses used more stringent methods for testing the null hypothesis, thereby increasing the robustness of the results.

## Results

### General

**Fig. 1:**
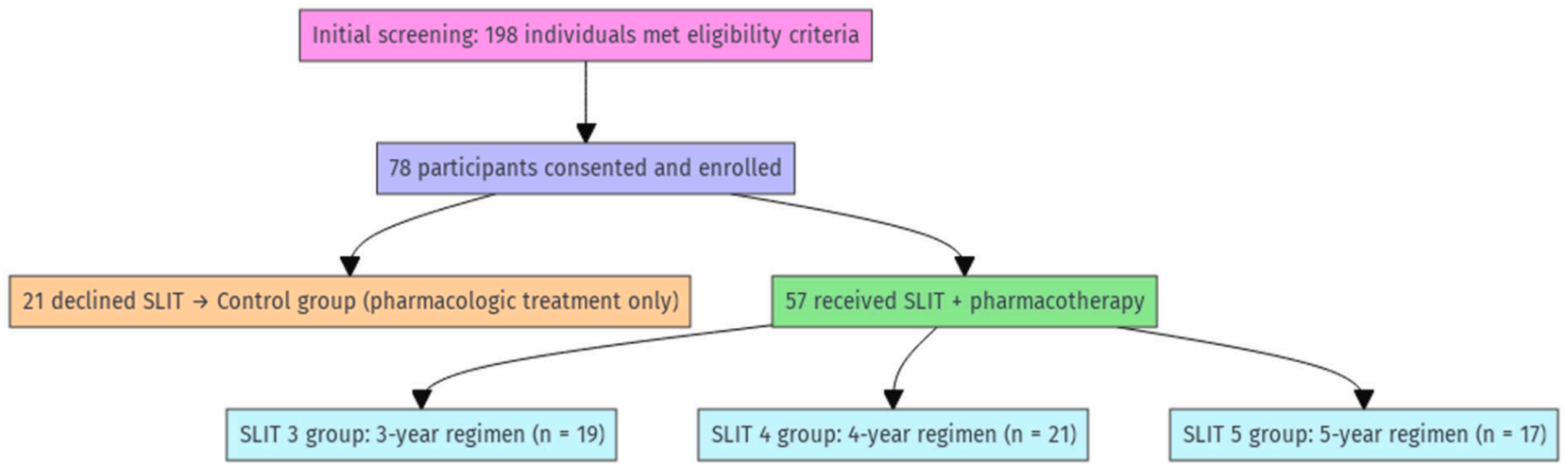
Flow diagram of the study design, group allocation, treatment duration, and 15-year follow-up (modified from reference 12 with permission from the authors). Of 198 individuals screened, 78 enrolled: 21 received pharmacological therapy alone (SLIT 0), and 57 received SLIT plus pharmacotherapy and were allocated to 3-, 4-, or 5-year regimens (n = 19, 21, and 17, respectively)

**Fig. 2:**
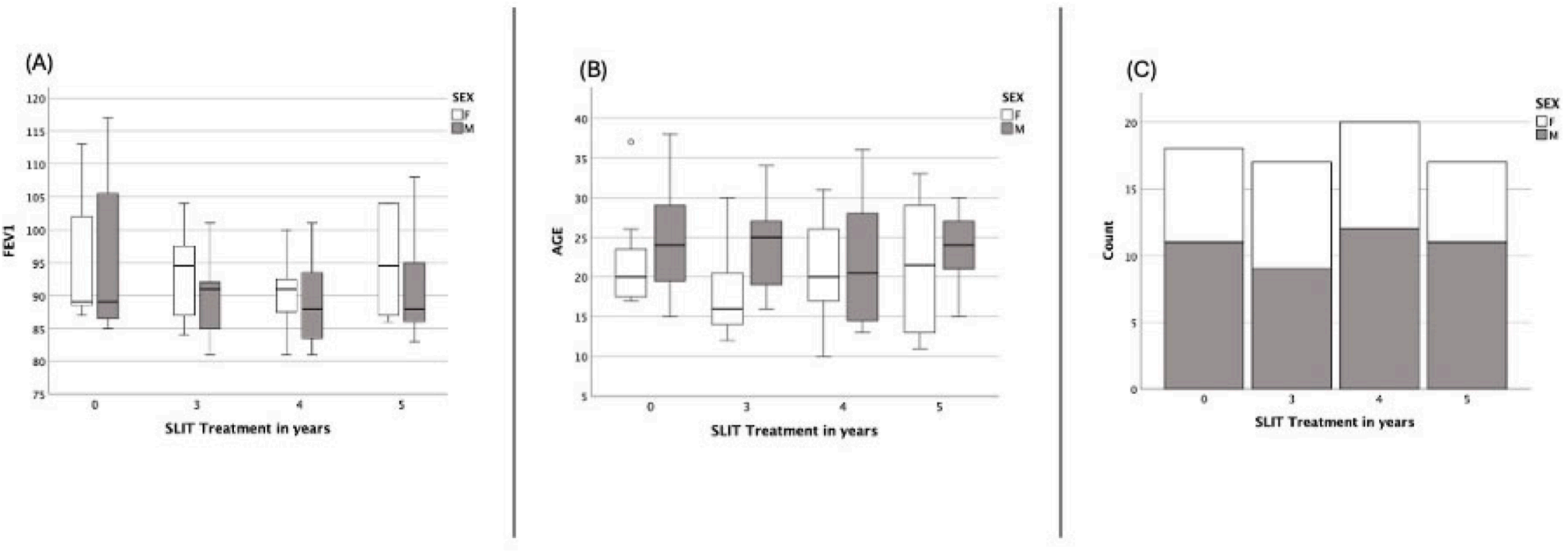
Baseline characteristics of the study population by treatment group. (A) Distribution of FEV1 (% predicted) across the control (SLIT 0) and SLIT 3, SLIT 4, and SLIT 5 groups. (B) Age distribution across treatment groups. (C) Number of participants in each treatment group. In panels A and B, box plots show the distributions for female and male participants; in panel C, stacked bars show sex-specific participant counts

During initial screening, 198 individuals met all eligibility criteria; of these, 78 participants (44 males and 34 females; mean age, 22.2 years) provided informed consent and underwent baseline assessments between September 1992 and February 1993. The study design is summarized in Fig. 1, Fig. 2. At study entry, the 4 groups had comparable demographic characteristics and baseline FEV1 values. During the 15-year follow-up, 19 participants discontinued the study: 5 each from the SLIT 3 and SLIT 4 groups and 9 from the control group. All discontinuations were attributed to protocol noncompliance, missed follow-up visits, or relocation. Ultimately, 59 participants completed the 15-year per-protocol evaluation (14 in SLIT 3, 16 in SLIT 4, 17 in SLIT 5, and 12 in the control group). Their baseline data did not differ significantly from those of patients who discontinued the study. Adherence to SLIT, assessed by measuring the residual volume in returned vials, consistently exceeded 80% in all SLIT groups. The proportions of male and female participants were comparable across treatment groups (control, SLIT 3, SLIT 4, and SLIT 5), with no statistically significant difference (=0.510, df = 3, p = 0.930). Similarly, neither FEV1 nor age differed significantly among treatment groups stratified by sex (FEV1: F_(7,64)_ = 1.268, p = 0.2802; age: F_(7,64)_ = 0.8838, p = 0.5244) (Table 2).

**Table 2:** Baseline FEV1 (% predicted) and age of patients with allergic rhinitis, stratified by sex and treatment group: pharmacotherapy alone (SLIT 0) or sublingual immunotherapy administered for 3, 4, or 5 years (SLIT 3, SLIT 4, and SLIT 5). Data are presented as the mean ± standard error of the mean (SEM), 95% confidence interval (CI), and minimum–maximum range

SLIT (Year)	Sex	Number	FEV_1_			Age		
			Mean ± SEM	95% CI	Min – Max	Mean ± SEM	95% CI	Min – Max
0	F	7	96 ± 4	86–105	87–113	22 ± 3	16–29	17–37
0	M	11	97 ± 4	88–105	85–117	25 ± 2	20–29	15–38
3	F	8	93 ± 2	87–99	84–104	18 ± 2	13–23	12–30
3	M	9	89 ± 2	85–94	81–101	24 ± 2	20–28	16–34
4	F	8	90 ± 2	86–95	81–100	21 ± 2	15–27	10–31
4	M	12	89 ± 2	85–93	81–101	22 ± 2	17–27	13–36
5	F	6	95 ± 4	86–104	86–104	22 ± 4	12–31	11–33
5	M	11	91 ± 2	86–97	83–108	24 ± 1	20–27	15–30

### Progression from rhinitis to asthma

**Fig. 3:**
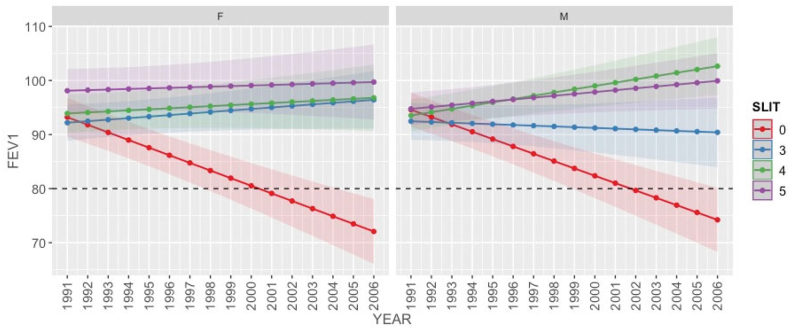
Model-estimated longitudinal FEV1 trajectories by sex and SLIT treatment duration. In the SLIT 4 and SLIT 5 groups, FEV1 improves over time in both sexes. In the SLIT 3 group, males show a declining trajectory, whereas females show a stable or slightly improving trajectory; the overall interaction involving sex was not statistically significant. Panels show female (F) and male (M) participants separately; colored lines represent SLIT 0, SLIT 3, SLIT 4, and SLIT 5, shaded bands indicate model uncertainty, and the dashed horizontal line marks the FEV1 threshold of 80% predicted

Persistent asthma (FEV1 < 80%) developed more frequently in the control group (7/12 patients, 58%) than in the SLIT groups, in which progression to asthma was significantly less frequent (SLIT 3: 1/14 patients, 7%, p = 0.009; SLIT 4: 1/16 patients, 6%, p = 0.004; SLIT 5: 1/17 patients, 6%, p = 0.003). The generalized linear mixed model (GLMM) showed no substantial violations of model assumptions, and the diagnostic findings were acceptable (Supplementary Figure 1). FEV1 trajectories were significantly affected by the YEAR × SLIT interaction (p < 0.001), indicating that the treatment effects increased over time (Table 3; Fig. 3).

**Table 3:** Fixed-effect estimates from the best-fitting linear mixed-effects model of longitudinal FEV1 values in patients with allergic rhinitis enrolled in 1991 and followed through 2006. The model compares pharmacotherapy alone (SLIT 0) with sublingual immunotherapy administered for 3, 4, or 5 years and reports chi-square statistics, degrees of freedom (df), p values, and significance levels for main effects and interactions.

Term	Chisq	Df	Pr(>Chisq)	Significance
YEAR	1.536	1	0.215	ns
SLIT	24.356	6	0.0004491	∗∗∗
SEX	2.158	4	0.7068	ns
AGE	0.877	1	0.349	ns
YEAR:SLIT	111.007	3	<2.2e-16	∗∗∗
YEAR:SEX	0.468	1	0.494	ns
SLIT:SEX	2.049	3	0.562	ns
YEAR:SLIT:SEX	5.204	3	0.157	ns
Total model vs null	0.000	51	1	ns

∗∗∗ p<0.001

No significant effects of AGE or SEX were detected, and no significant interactions involving these variables were observed (Table 3; p > 0.05). However, the partial-effects plot (Fig. 3) indicates that males in the SLIT 3 group had a response trajectory over time that differed from that of females.

### Skin sensitization

During the 15-year follow-up, all patients in the control group developed at least 1 new skin sensitization. By contrast, at the end of the study, new sensitizations were observed in 3/14 patients (21.4%) in the SLIT 3 group, 2/16 (12.5%) in the SLIT 4 group, and 2/17 (11.7%) in the SLIT 5 group. The between-group difference became statistically significant in year 6 (p = 0.03, test).

## Discussion and conclusions

Respiratory allergic diseases, particularly allergic rhinitis, often precede asthma onset, a phenomenon known as the "allergic march".^18^ Several studies have reported that HDM SLIT can reduce the incidence of asthma among treated patients compared with control groups.^19^, ^20^, ^21^, ^22^

The present study differs from the existing literature in several respects, including the longer observation period, the different treatment durations (SLIT 3, SLIT 4, and SLIT 5), and the assessment of AIT effects on lung function and asthma development. The principal limitation of this study was the absence of double blinding; however, this limitation is partly offset by the long trial duration and, retrospectively, by the increased recognition of evidence from nonblinded real-world studies.^23^, ^24^, ^25^

The first observation is that progression to asthma (FEV1 < 80%) occurred in 58% of patients with rhinitis who received pharmacological therapy alone. By contrast, only 1 patient in each SLIT group showed a similar decline in FEV1, regardless of treatment duration (3, 4, or 5 years), even 10–12 years after SLIT had ended. In addition, although the difference was not significant, males tended to respond less favorably than females after only 3 years of SLIT (Fig. 3).

The ability of AIT to maintain FEV1 values above 80% is particularly important because an FEV1 above 80% of the predicted value indicates minimal airway narrowing, good symptom management, and well-controlled disease. These observations are consistent with previous studies in which early allergen immunotherapy reduced the onset of asthma in children with allergic rhinoconjunctivitis.^26^^,^^27^ The role of SLIT in preventing asthma supports the view that allergen immunotherapy is the only disease-modifying treatment for respiratory allergies and is endorsed by clinical guidelines.^28^ Moreover, meta-analyses confirm that multi-year SLIT, including HDM-specific formulations, reduces the development of new sensitizations and the risk of asthma^29^, ^30^, ^31^.

Further analysis of the original data using an advanced GLMM demonstrated a significant interaction between SLIT and treatment duration (p < 0.001), suggesting that the protective effect of SLIT strengthens over time. This finding is consistent with clinical trials showing that 4-year SLIT regimens provide more durable efficacy than shorter courses. Notably, the present study extends the documented duration of benefit, with effects persisting for at least 12 years after treatment, potentially because of immunological tolerance mediated by regulatory T cells and allergen-specific IgG and IgA production. These findings provide additional real-world support for registry-based evidence of the long-term benefit of SLIT in preventing asthma onset.^30^

Another finding in our cohort was the potentially sex-dependent efficacy of the 3-year regimen. Although the overall effects of sex were not significant, partial-effects plots suggested that males receiving SLIT for 3 years responded less favorably than females. This difference warrants further investigation. One possible explanation is that sex hormones modulate regulatory T-cell induction or immunoglobulin class switching during the critical early treatment period.^32^ Future randomized controlled trials should be adequately powered to test these hypotheses.

New skin sensitizations developed in 100% of control participants over 15 years but in only 11–21% of SLIT recipients (p = 0.03 by year 6). These results are consistent with several randomized and observational studies showing that SLIT significantly reduces the rate of new sensitization. This finding indicates that the immunomodulatory capacity of SLIT extends beyond clinical symptom relief to modification of allergic sensitization patterns.

Although the open-label design may theoretically introduce bias, this risk was substantially mitigated by the structure of the study. The initial treatment choice was made after full and transparent disclosure, and all patients were free to select among the previously described treatment strategies, each of which was guideline-endorsed and clinically effective, making substantial selection bias unlikely. Moreover, outcomes were assessed consistently and without modification throughout the follow-up period, ensuring complete data collection for at least 15 years. Together, these features help mitigate the methodological limitation of the open-label design, making any residual bias unlikely to compromise interpretation of the results.

Importantly, participant adherence exceeded 80%, and attrition was balanced across the SLIT groups, indicating that substantial bias due to noncompliance was unlikely. Baseline equivalence in demographic characteristics, FEV1, and sex distribution strengthened internal validity. Nevertheless, several limitations warrant consideration. First, the sample size in each SLIT arm was modest (n = 14–17 completers), limiting the power to detect subgroup effects. Furthermore, the pharmacological context evolved over the 15-year follow-up as guideline-recommended treatments changed; however, no treatment changes were adopted in this study because the new guidelines were introduced only in 2020.^33^ Another potential source of bias is that the definition and treatment of asthma evolved over time, but the parameters established at study onset were applied consistently through the final assessment. Against this background, the sustained divergence in FEV1 trajectories between the SLIT and control groups suggests a genuine disease-modifying effect.

This long-term follow-up study demonstrates that a 3–5-year course of SLIT substantially decreases the risk of progression from rhinitis to asthma, with benefits persisting for at least 12 years after treatment. Regimens lasting 4–5 years appeared optimal for sustained benefit. Clinical guidelines, including ARIA, EAACI, and GINA, should increasingly recommend extended SLIT for patients with allergic rhinitis who are at high risk of developing asthma. The differential response by sex in the SLIT 3 group requires further investigation and suggests the potential value of personalized SLIT strategies. Overall, these findings reinforce the role of SLIT as a safe, patient-friendly, disease-modifying therapy capable of preventing the allergic march.

## Authors’ consent for publication

All authors approve publication.

## Author contributions

MM, DB, GWC, SF, BB, GP writing and approving; AM statistical analysis.

## Ethics approval

Not applicable.

## Use of generative intelligence AI

Nothing to disclose.

## Funding

None.

## Availability of data and materials

At request.

## Competing interests

None for any authors.
