# Supplementary material for: Long-term effects of sublingual immunotherapy (SLIT) in preventing the development of asthma in allergic rhinitis: Revision of 15-year follow-up
Source: World Allergy Organ J. 2026 Jul 28;19(8):101434. doi: 10.1016/j.waojou.2026.101434 (PMC13446181; doi:10.1016/j.waojou.2026.101434)
Supplement: Supplementary file 1 [file mmc1.docx]

# Supplementary 1

**Supplementary Figure 1. Diagnostic plots for the selected generalized linear mixed** model of longitudinal FEV1, used to assess model assumptions and goodness of fit. Panels display the posterior predictive check, residual linearity, homogeneity of variance, collinearity, normality of residuals, and normality of random effects.
